# Microbial Fermentation of Industrial Rice-Starch Byproduct as Valuable Source of Peptide Fractions with Health-Related Activity

**DOI:** 10.3390/microorganisms8070986

**Published:** 2020-06-30

**Authors:** Elena Babini, Danielle Laure Taneyo-Saa, Annalisa Tassoni, Maura Ferri, Axel Kraft, Jürgen Grän-Heedfeld, Karlheinz Bretz, Aldo Roda, Elisa Michelini, Maria Maddalena Calabretta, Fabien Guillon, Davide Tagliazucchi, Serena Martini, Lorenzo Nissen, Andrea Gianotti

**Affiliations:** 1 Department of Agricultural and Food Sciences (DiSTAL), Alma Mater Studiorum—University of Bologna, V.le Fanin 44, 40127 Bologna, Italy; elena.babini2@unibo.it (E.B.); danielle.taneyosaa@studio.unibo.it (D.L.T.-S.); lorenzo.nissen@unibo.it (L.N.); 2 Department of Biological Geological and Environmental Sciences (BIGeA), Alma Mater Studiorum—University of Bologna, Via Irnerio 42, 40126 Bologna, Italy; annalisa.tassoni2@unibo.it (A.T.); maura.ferri@unibo.it (M.F.); 3 Department of Civil, Chemical, Environmental and Materials Engineering (DICAM), Alma Mater Studiorum-University of Bologna, Via Terracini 28, 40131 Bologna, Italy; 4 Fraunhofer Institute for Environmental, Safety and Energy Technology (UMSICHT), Osterfelder Str. 3, 46047 Oberhausen, Germany; axel.kraft@umsicht.fraunhofer.de (A.K.); juergen.graen-heedfeld@umsicht.fraunhofer.de (J.G.-H.); karlheinz.bretz@umsicht.fraunhofer.de (K.B.); 5 Department of Chemistry “Giacomo Ciamician” (CHIM), Alma Mater Studiorum—University of Bologna, Via Selmi 2, 40126 Bologna, Italy; aldo.roda@unibo.it (A.R.); elisa.michelini8@unibo.it (E.M.); maria.calabretta2@unibo.it (M.M.C.); 6 Sterlab, Cell Culture Laboratory, Ch. St-Bernard 2720, 06224 Vallauris Cedex, France; fguillon@sterlab.com; 7 Department of Life Sciences (DSV), University of Modena and Reggio Emilia, Via Amendola 2, 42122 Reggio Emilia, Italy; davide.tagliazucchi@unimore.it (D.T.); serena.martini@unimore.it (S.M.)

**Keywords:** rice byproduct, rice protein, protein hydrolysates, *Lactobacillus* spp., bioactive peptides

## Abstract

The rice-starch processing industry produces large amounts of a protein-rich byproducts during the conversion of broken rice to powder and crystal starch. Given the poor protein solubility, this material is currently discarded or used as animal feed. To fully exploit rice’s nutritional properties and reduce this waste, a biotechnological approach was adopted, inducing fermentation with selected microorganisms capable of converting the substrate into peptide fractions with health-related bioactivity. Lactic acid bacteria were preferred to other microorganisms for their safety, efficient proteolytic system, and adaptability to different environments. Peptide fractions with different molecular weight ranges were recovered from the fermented substrate by means of cross-flow membrane filtration. The fractions displayed in vitro antioxidant, antihypertensive, and anti-tyrosinase activities as well as cell-based anti-inflammatory and anti-aging effects. In the future, the peptide fractions isolated from this rice byproduct could be directly exploited as health-promoting functional foods, dietary supplements, and pharmaceutical preparations. The suggested biotechnological process harnessing microbial bioconversion may represent a potential solution for many different protein-containing substrates currently treated as byproducts (or worse, waste) by the food industry.

## 1. Introduction

Rice is a staple food crop for about half of the world’s population. Industrial processing produces large amounts of broken kernels, which are mainly converted into powder and crystal starch. During this conversion process, most of the protein fraction (approximately 60%–85%) remains in a residual product which is currently discarded or underutilized as animal feed. In recent years, enzymatic proteolytic modification has been proposed as an efficient approach to improve the functional properties and biological activities of this proteinaceous byproduct and allow its exploitation [1,2,3,4,5,6,7]. In particular, Ferri and coworkers produced, starting from this substrate, enzymatic hydrolysates with a broad spectrum of biological activities (antioxidant, anti-tyrosinase, antihypertensive, and anti-inflammatory) [6]. These results are consistent with the results previously obtained on rice endosperm proteins [8,9,10,11], confirming the presence, in cereal storage proteins, of bioactive peptides with a potential role in the prevention of chronic diseases [12,13,14]. Microbial proteolysis, also known as microbial fermentation, is an alternative, less expensive method for producing bioactive peptides from protein substrates. The major advantage of using microbes instead of enzymes is that appropriately used microorganisms can not only break down proteins into peptides and free amino acids, but they can also remove hyper-allergenic or anti-nutritional factors that may be present (e.g., trypsin inhibitors, phytate, oligosaccharides such as raffinose and stachyose, and saponins in legumes). Over the past two decades, besides traditionally fermented dairy and cereal products such as Koji or sourdough bread, microbial fermentation has been applied to other food matrices as an effective way to generate peptides with different bioactive properties [15,16,17,18,19,20]. However, this approach is still largely unexplored. To date, only one paper [4] refers to the enhancement of antioxidant properties of rice-starch byproduct obtained with microbial fermentation by *Bacillus pumilus* AG1. The goal of the current study was to further investigate the feasibility of microbial fermentation by developing a process to produce hydrolysates from rice-starch byproduct (hereafter referred to as BR) and testing the bioactivity of resulting peptides. The Lactic Acid Bacteria (LAB) were preferred to other microorganisms for their safety, efficient proteolytic system, and adaptability to different environments [21,22,23]. After separation by cross-flow membrane filtration, the peptide fractions were tested for bioactivity (antioxidant, antihypertensive, anti-tyrosinase, anti-inflammatory, and anti-aging properties), in order to evaluate their potential as health-promoting ingredients in functional foods, dietary supplements, and pharmaceutical or cosmetic preparations.

## 2. Materials and Methods

### 2.1. Materials

BR was obtained from Amideria il Cervo Srl (Monterenzio, Italy). Pre-cast gels, the MW marker for sodium dodecyl sulfate-polyacrylamide gel electrophoresis (SDS-PAGE), bovine serum albumin (BSA), and related reagents were obtained from Bio-Rad (Hercules, CA, USA). Additional reagents were obtained from Sigma (Saint Louis, MO, USA), Merck (Darmstadt, Germany) or Oxoid (Altrincham, England). The Elastin and Collagen I Elisa Kits were obtained from Antibodies-on-line (Atlanta, GA, USA). Normal Human Fibroblasts were isolated from human foreskin at Sterlab facilities (Vallauris, France). FuGENE^®^HD and plasmid pGL4.32[luc2P/NF-κB-RE/Hygro] were obtained from Promega (Madison, WI, USA). The plasmid pcDNA.3.1-mcherryPRET9, has been previously described [6]. Embryonic kidney HEK293 cells came from American Type Culture Collection (Manassas, VA, USA). Dulbecco Modified Essential Medium (DMEM), Fetal Bovine Serum (FBS), L-glutamine, and penicillin-streptomycin came from GE Healthcare Life Sciences (Buckinghamshire, UK).

### 2.2. Microorganisms

Eleven *Lactobacillus* strains were considered for BR fermentation. Seven strains, *Lactobacillus plantarum* 82 (Lp82), *Lb. plantarum* 325 (Lp325), *Lb. rhamnosus* C1122 (LrC1122), *Lb. casei* lbcd (Lclbcd), *Lb. fermentum* MR13 (LfMR13), *Lb. plantarum* 6BHI (Lp6BHI), and *Lb. rhamnosus* C249 (LrC249), were from the collection of the Department of Agricultural and Food Sciences of the University of Bologna, Italy. The other five, Lb. rhamnosus PRRH (LrPRRH), *Lb. fermentum* PRFE (LfPRFE), *Lb. plantarum* PRPL (LpPRPL), and *Lb. bulgaricus* PRBU (LbPRBU), were kindly provided by Principium SA (Switzerland). Lactic acid bacteria (LAB) counts were performed on MRS (Oxoid, Thermo Fisher Scientific, USA) agar medium after incubation for at least 24 h at 37 °C in jars with an anaerobiosis catalyst (Oxoid, Thermo Fischer Scientific, USA).

### 2.3. Microbial Fermentation of BR

Bacterial fermentation of BR was performed in small-scale (50 mL) and large-scale (1 L) volumes at 37 °C with regular rotary shaking at 150 rpm for 72 h. In order to increase bacterial growth in BR, a fermentation substrate (FS, made of: glucose 7 g/L, fructose 7 g/L, maltose 7 g/L, sodium acetate 5 g/L, K_2_HPO_4_ 2.5 g/L, MgSO_4_ 0.2 g/L, MnSO_4_·4H_2_O 0.05 g/L, FeSO_4_·4H_2_O 0.01 g/L, and Tween 80 1 g/L, pH 5.6) was used as medium enrichment as explained below. The LAB pre-inoculum was prepared from 1:1 volume overnight bacterial culture, grown in MRS broth. The cells were then harvested by centrifugation at 12,000 g for 10 min at 4 °C, washed twice with 20 mM phosphate buffer (pH 5.6) and resuspended in: (a) BR; (b) BR with FS; or (c) BR5X (BR concentrated 5 times) with FS. The initial load of inoculated LAB was approximately 10^9^ CFU/mL. Controls consisted of each kind of substrate (a, b, and c), without inoculation. Samples for protein quantification, SDS-PAGE, and bioactivity analysis were made by the supernatants collected after centrifugation of culture aliquots, at 8000 g, for 30 min at 4°C. Samples were stored at −80 °C until use.

In order to check the predominance of inoculated strains in large scale fermented samples after 72 h of incubation (BR + FC samples to be further characterized for their bioactive properties), microbial DNA from the samples stored at −80 °C was extracted with a commercial kit, namely NucleoSpin Food (Macherey Nagel, Duren, Germany) following supplier’s instruction. DNA concentration and purity was then checked spectrophotometrically with a BioDrop (Biodrop, Cambridge, UK). PCR on extracted samples was performed with two different reactions employing different enzymes, but the same primer pairs related to generic *Lactobacillus* spp. 16S-rDNA V3-V4, named Lac1 (forward:5′-GCAGCAGTAGGGAATCTTCCA-3′ and reverse: 5′-GCATTYCACCGCTACACATG-3′) [24] (Eurofins Genomics, Ebersberg, Germany). The first reaction (R1) employed AmpliTaq (Applied Biosystems, Thermo Fisher Scientific, Foster City, CA, USA), a random-use polymerase, while the second (R2) used SuperFi Platinum Taq (Invitrogen, Thermo Fischer Scientific, Carlsbad, CA, USA), a polymerase with 300 times higher fidelity than AmpliTaq, that can stand very GC-rich and low copy number templates, as those derived from our putative target. Both reactions were performed in 25 uL volume, that for R1 contained: 200 nM of each primer, 1 × Taq buffer, 1.5 mM of MgCl_2_, 0.2 mM of dNTPs mix, 0.625 U/rx of AmpliTaq, and 2 ng of DNA. R2 contained: 500 nM of each primer, 1 × SuperFI buffer, 1 x SuperFi GC enhancer, 0.2 mM of dNTPs mix, 0.5 U/rx of SuperFI Platinum Taq, and 1 ng of DNA. Lac1 amplicons were obtained using a ProFlex PCR System apparatus (Applied Biosystems, Thermo Fischer Scientific, USA) set as follows: For R1, a denaturation stage at 95 ℃ for 4 min, a cycling stage repeated 30 times (95 ℃ for 30 s, 60 ℃ for 30 s, 72 ℃ for 1 min), and an extension stage at 72 °C for 7 min. For R2, a denaturation stage at 98 ℃ for 2 min, a cycling stage repeated 30 times (98 ℃ for 20 s, 72 ℃ for 30 s), and an extension stage at 72 °C for 5 min. Amplicons ran electrophoretically on a 2 % TAE/agarose gel (TopVision Agarose, Thermo Fisher Scientific, Waltham, MA, USA) and were stained by 0,01 % GelRed (Biotium Inc., Fremont, CA, USA). 6 μL/lane of 100 bp Ladder (Invitrogen, Thermo Fisher Scientific, USA) was employed.

### 2.4. Protein Quantification and SDS-PAGE

Protein concentration was estimated by Lowry assay [25]. Briefly, the reaction solution was prepared by mixing: 50 mL of Na_2_CO_3_ 2% (w/v) solution in NaOH 0.1 N, 0.5 mL of CuSO_4_·5H_2_O 1% (w/v) solution in water, and 0.5 mL of potassium sodium tartrate·4H_2_O 2% (w/v) solution in water. An aliquot of the reaction solution (2 mL) was mixed with 0.1 mL of sodium deoxycholate 10% (w/v) solution in water, 0.8 mL of water, 0.1 mL of sample and, after 5 min, 0.2 mL of 50% (v/v) Folin–Ciocalteau reagent solution in water. After 30 min incubation at room temperature, the absorbance of the solution was measured at 750 nm. The calibration was performed with BSA solutions in concentrations ranging from 0 to 25 μg/mL. Protein pattern analysis was performed using hand-cast 14% Tris SDS-polyacrylamide gels and pre-cast 10%–20%, Tris-Tricine SDS-polyacrylamide gels on Mini-PROTEAN^®^ equipment from Bio-Rad (Hercules, CA, USA). The Precision Plus Protein Standard and Polypeptide SDS-PAGE MW Standard, from the same company, were used as markers.

### 2.5. Fractionation of Microbial Protein Hydrolysates

The hydrolyzed peptides in the liquid supernatants resulting from large-scale microbial digestion were fractionated using a Sartoflow Slice 200 Benchtop CrossFlow System (Sartorius Stedim Biotech GmbH, Göttingen, Germany). Four polyethersulfone (PESU) membranes with cut-offs of 0.2 µm (0.02 m^2^), 8, 5, and 1 kDa (0.04 m^2^) were used in sequence for separation, leading to five samples of 50 mL each: 4 retentates (R0.2, R8, R5 and R1) and the 1 kDa permeate (P1). Fractions were lyophilized and stored at −18 °C. For bioactivity analysis, they were resuspended in water and the pH was adjusted to 8 with NaOH.

### 2.6. In Vitro Antioxidant Activity Evaluation

The antioxidant activity was assayed with 2,2-diphenyl-1-picrylhydrazyl (DPPH) and 2,2-azino-bis(3-ethylbenz-thiazoline-6-sulfonic) acid (ABTS) decolorimetric assays, according to previously published methods [26,27]. In particular, for the DPPH assay the sample was added in a ratio of 1:10 (v/v) to a 100 μM DPPH solution in methanol. The mixture was shaken and incubated for 30 min at room temperature in the dark, and after that the absorbance was measured at 517 nm. The value was corrected with a water blank. For the ABTS assay equal volumes of ABTS (7 mM in 20 mM sodium acetate, pH 4.5) and potassium persulfate (2.45 mM in water) were mixed and incubated for 12–16 h at room temperature in the dark until the solution reached a stable oxidative state. The solution was then diluted with 20 mM sodium acetate (pH 4.5) to achieve an absorbance of 0.700 ± 0.01 at 734 nm. The sample was added in a ratio of 1:10 (v/v) to the ABTS^+^ solution and shaken. After the solution had been left for 30 min at room temperature in the dark, its absorbance at 734 nm was read. The value was corrected with a water blank. All assays were performed in triplicate, with at least three technical replicates (n = 3 ± SD). Results were expressed as mg of ascorbic acid (AA) equivalents/L (mg AA eq/L), or mg AA equivalents /g of proteins (mg AA eq/g prot), on the basis of a dose-dependent calibration curve (0–2 µg of AA).

### 2.7. Angiotensin-Converting Enzyme (ACE)-Inhibitory Activity Measurement

The ACE-inhibitory activity was estimated using the spectrophotometric assay of Ronca-Testoni [28] using as substrate the tripeptide N-[3-(2-furyl)-acryloyl]-L-phenylalanyl-glycyl-glycine (FAPGG), as previously described [29]. The assay was carried out in the presence of different amounts of the peptide fractions to calculate their IC50 values (IC50, expressed as μg of peptides/mL, is the concentration of peptides required to inhibit 50% of the enzymatic activity). The IC50 values were determined using nonlinear regression analysis, fitting the spectrophotometric data with the log (inhibitor) vs. response model generated by GraphPad Prism 6.0 (GraphPad Software, San Diego, CA, USA).

### 2.8. In Vitro Anti-Tyrosinase Activity Determination

Anti-tyrosinase activity was measured using an optimized tyrosinase-inhibition assay [6]. The kinetics of brown color formation were evaluated at 490 nm in a reaction containing 10 U of tyrosinase and 2 mM L-DOPA in the presence of the sample. All assays were performed with at least three technical replicates (*n* = 3 ± SD), and the results were expressed as mg of kojic acid (KA, a well-known tyrosinase inhibitor) equivalents/g of proteins (mg KA eq/g prot) by a dose-dependent calibration curve (1–10 μg KA).

### 2.9. Cell-Based Assay for Anti-Inflammatory Activity Evaluation

A dual-color reporter assay for anti-inflammatory activity was performed using human embryonic kidney HEK293 cells grown in DMEM high glucose (4.5 g/L), supplemented with 10% fetal bovine serum, 2 mM L-glutamine, 50 U/μL penicillin, and 50 μg/mL streptomycin. The assay was performed as previously described [30], with slight modifications. Briefly, HEK293 cells were plated in 24-well plates the day before transfection, at a density of 8 × 10^4^ cells per well, in 500 µL of complete growth medium. Cells were co-transfected with plasmid pGL4.32[luc2P/NF-κB-RE/Hygro] and pcDNA.3.1-mcherryPRET9 using FuGENE^®^HD according to the manufacturer’s instructions and incubated for 24 h at 37 °C with 5% CO_2_. Then the cells were treated for 5 h with 500 μL of fresh medium containing sample supernatants (1:10 dilutions in sterile H_2_O) and 5 ng/mL tumor necrosis factor α (TNFα). Subsequently, the cells were detached with trypsin EDTA 1X in PBS, and the pellets were resuspended in 100 μL PBS 0.1 M at pH 7.5 and transferred to a black 96-well microplate. After the injection of 100 μL substrate D-Luciferin 1.0 mM pH 5.5, bioluminescence (BL) was measured with a Varioskan™ Flash Multimode Reader (Thermo Fisher), which acquired BL signals with band-pass green and red filters. Corrected values were obtained using an Excel spreadsheet (ChromaLuc Calculator) to correct bioluminescence signals according to cell viability. All assays were performed in triplicate, with at least three technical replicates (*n* = 3 ± SD).

### 2.10. Cytotoxicity and Anti-Aging Tests

Cytotoxicity and anti-aging tests were performed on Normal Human Fibroblast (NHF) monolayer cultures. For the former, cells were systemically exposed to samples with concentrations of 0.01% or 0.1% from stock solution for 72 h at 37 °C with 5% CO_2_. The cell viability was expressed as a percentage of the mean negative control cells: cell viability above 50% indicated the tested substance was non-toxic. A positive control was performed using 0.5% SDS solution. The anti-aging evaluation was performed by comparing the expression level of human elastin and human collagen in the supernatants to the basal expression level in supernatants of untreated fibroblasts. Vitamin C was used as a positive control for the anti-aging test.

### 2.11. Statistical Analysis

Results were calculated from the mean of at least two values. An ANOVA with Tukey’s multiple comparison test was used to assess the statistical significance of differences among several means (“R” software, R Core Team, Vienna, Austria 2017; version 3.4.2).

## 3. Results and Discussion

### 3.1. BR Protein Content and Pattern

The BR was a heterogeneous mixture containing 7.5% solids (average value from eleven samples) in a liquid phase, with a total protein content of 6.0 ± 4.6 g/L [6]. Proteins with MWs between 5 and 200 kDa (Supplementary Materials, Figure S1) were more concentrated in the solid fraction; less than 5% of the total proteins were found in the liquid phase [6].

### 3.2. Lactobacillus Strain Selection

Eleven LAB strains and their control (not inoculated BR) were screened for hydrolytic activity towards the substrate and for the resulting hydrolysates’ antioxidant activity, in small scale (50 mL) experiments. On the basis of the results of these preliminary tests (Supplementary Materials, Figure S2), the three best-performing strains (LrC1122, Lp82, and LrPRRH), as well as the not inoculated control, were selected for optimization of fermentation conditions, in 50 mL cultures. 

### 3.3. Optimization of BR Fermentation Conditions

**Figure 1:**
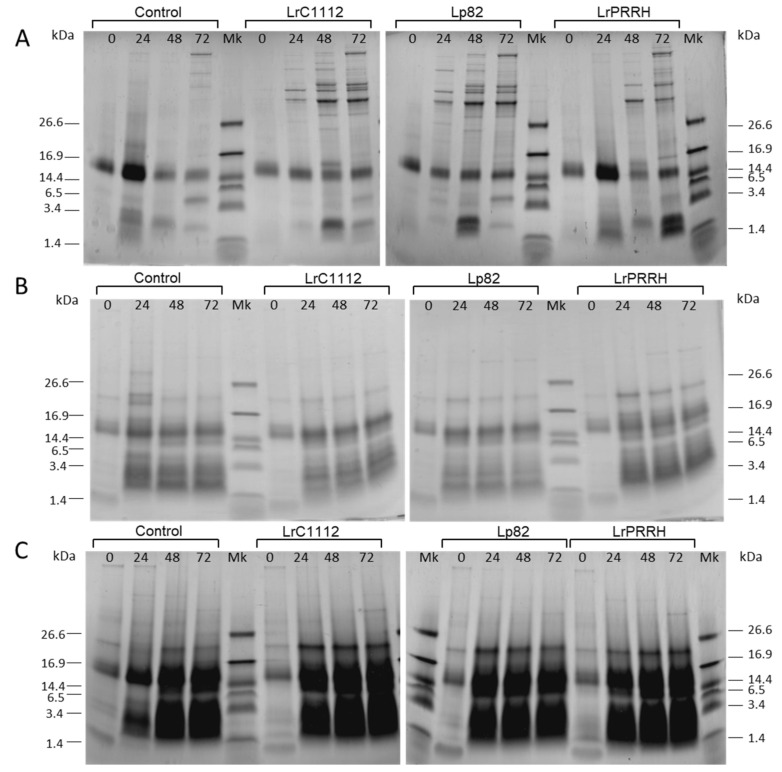
Tricine SDS-PAGE (at incubation times 0, 24, 48, and 72 h), of rice protein byproduct (BR) not inoculated (control samples), and of BR incubated with strains LrC1122, Lp82, and LrPRRH, in three different experimental conditions (50 mL scale): (**A**) standard conditions; (**B**) with addition of fermentation substrate (FS); (**C**) with rice protein byproduct containing the solid fraction concentrated five times (BR5X), and addition of FS. Equal volumes of samples were loaded on gel.

Fermentation conditions were optimized for growth media, whole-cell/substrate ratio, and incubation time to achieve the highest hydrolytic and antioxidant activities towards the substrate. In particular, a sugar- and salt-enriched medium without nitrogen sources (FS) and a substrate rich in solid material (BR5X) were evaluated against BR, considered as standard. The pH, the number of viable LAB cells, protein concentration, antioxidant activity, and protein pattern were analyzed at 0, 12, 24, 48, and 72 h incubation times. Results are reported in Table 1 and Figure 1. In not inoculated samples (control samples at different times), LAB cells regularly grew, increasing their load of around one Log cycle/g after 72 h. On the other hand, the LAB inoculated samples did not evidence a similar growth with the exception of the LrPRRH strain in BR5X + FS, that reached the highest microbial load of around 10.8 CFU/g, after 72 h incubation time. Furthermore, differently from what was observed in BR samples, a pH decrease over time was observed in the other two growth conditions tested (BR with FS and BR5X with FS). The pH decrease was stronger in the LAB inoculated samples after 24 h incubation time, with respect to the not inoculated (BR) samples, particularly in the presence of the enriched substrate (BR5X with FS). Apparently, the addition of FS to the BR medium positively influenced the metabolic efficiency of selected strains, providing a pH decrease, already in the first 24 h, greater than that induced by naturally occurring microbiota. At 72 h, the soluble protein concentrations reached the maximum for both control (not inoculated) and inoculated samples, in all three growth conditions. The protein concentration ranged from 1.04 to 1.82 g/L under normal conditions, from 3.32 to 4.15 g/L in the presence of FS, and from 4.50 to 5.54 g/L in the presence of FS and BR5X with FS. The antioxidant activity of hydrolysates, measured by ABTS assay, reached the highest value after 72 h. The highest activity for both the control and inoculated samples occurred under standard growth conditions (values from 92.01 mg AA eq/L to 104.38 mg AA eq/L). In the other two conditions, the activity levels were variable. For LrC1122 and LrPRRH, the highest values were observed without the enriched substrate (66.49 mg AA eq/L and 79.80 mg AA eq/L, respectively), whereas for Lp82 and the control sample, the highest activity was obtained in the presence of BR5X and FS (56.54 mg AA eq/L and 59.30 mg AA eq/L, respectively). The protein pattern in the starting material, as detected by Tricine SDS-PAGE, consisted of a predominant band with MW between 14.4 and 16.9 kDa and a smeared band made of polypeptides with MW lower than 3.4 kDa. In standard growth conditions, higher-MW proteins were released from the solid phase during incubation, and were only partially degraded after 72 h (Figure 1A). In the other conditions, the concentrations of proteins and peptides with MW between 14.4 and 16.9 kDa and those with MW below 6.5 kDa strongly increased during incubation, indicating their release from the solid matrices due to hydrolysis by enzymes from inoculated or endogenous bacteria (Figure 1B,C). The results of these small-scale experiments indicate that FS-enriched media and a 72-h incubation time should be used to promote the hydrolytic activity of inoculated LAB. In these conditions, a significant (three- to four-fold) increase in protein/peptide concentration was observed with respect to the standard condition, which could lead to the quantitative and qualitative recovery of peptides with different bioactivities. The media with substrate enrichment were discarded in spite of a further increase in protein/peptide concentration (about 1.3 times), because they were less suitable in terms of time and cost for the large-scale process.

### 3.4. Characterization of the Large-Scale Process Hydrolysates

Large-scale fermentation (1 L) was performed under the same conditions as the small-scale experiments described above. The BR with the three selected LAB strains was incubated in the presence of FS at 37 °C for 72 h. Protein content, antioxidant activity, pH, protein pattern, and microbial counts were assayed at 0, 24, 36, and 72 h of incubation (Supplementary Materials, Table S1 and Figure S3). In order to assess the predominance of inoculated strains, a molecular characterization of fermented large-scale samples was performed with two different reactions employing different enzymes which confirmed the success of selected strains against naturally occurring LAB after a 72 h fermentation in BR + FS substrate (Supplementary Materials, Figure S4). The collected data confirmed the results obtained in the small-scale experiments: maximum protein concentration (3.58 g/L, 4.25 g/L, and 3.93 g/L, for LrC1122, Lp82, and LrPRRH strains, respectively) and antioxidant activity (50.59 mg AA eq/L, 44.93 mg AA eq/L, and 53.75 mg AA eq/L, for LrC1122, Lp82, and LrPRRH strains, respectively) were reached after 72 h of incubation. Accordingly, the 72-h samples were considered for separation of peptide fractions.

### 3.5. Fractionation of Hydrolysates

The hydrolysates obtained from the 1 L scale of Lp82, LrC1122, and LrPRRH fermentation trials were fractionated by crossflow filtration. The remaining particles in the liquid supernatant, obtained by centrifugation, were removed by the crossflow filtration using a membrane with a cut-off of 0.2 µm. The permeate was further processed using membranes with cut-offs of 8, 5, and 1 kDa. The code (A, B, C, for LrC1122, Lp82, and LrPRRH strains; numbers 1 to 5 for separation fractions R0.2, R8, R5, R1, and P1), filtration loss (%), concentration factor (mL/mL), and dry matter (g/L) of all the fractions are reported in Table 2. The volume and the weight of each sample at each filtration step were recorded for the volume and mass balance. The average volumetric filtration loss for all steps was 3.48%.

### 3.6. Biological Activity of Peptide Fractions

**Figure 2:**
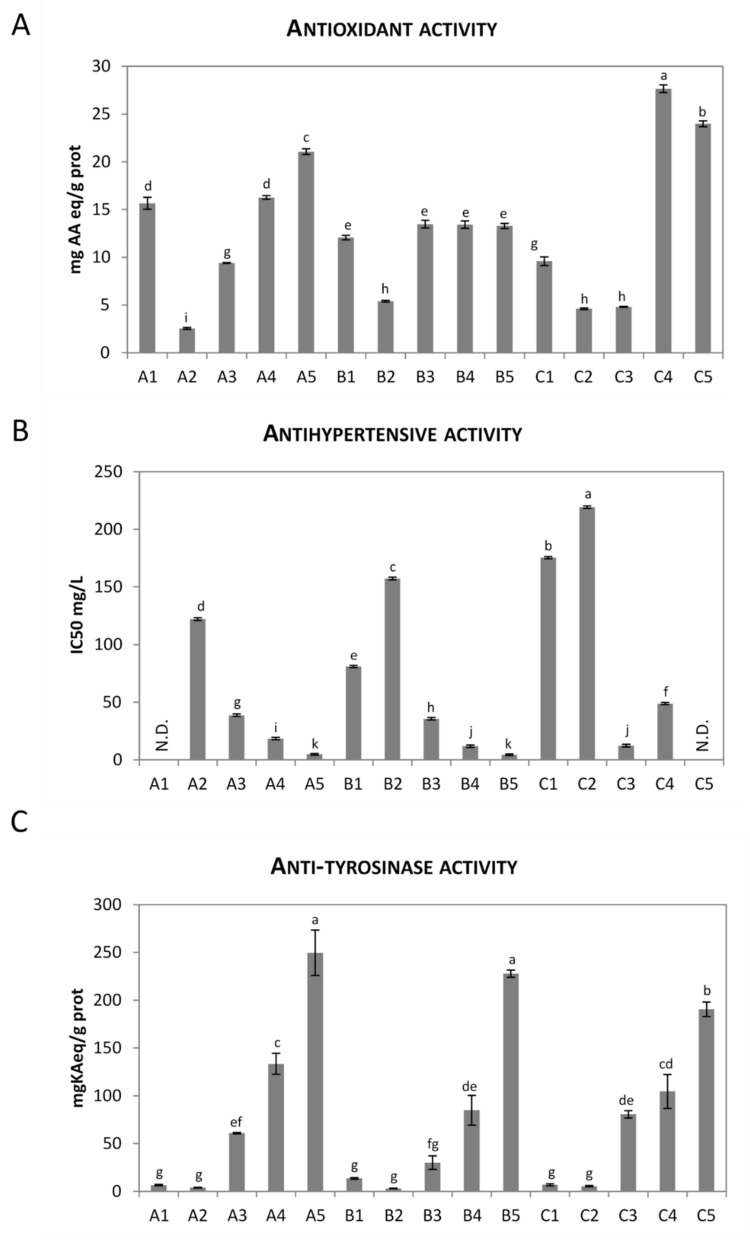
Antioxidant activity by ABTS assay (mg AA eq/g of proteins, **A**), antihypertensive activity IC50 (mg/L, **B**), and anti-tyrosinase activity (mg KAeq/g, **C**), of peptide fractions 1 to 5 (1 = fraction with particle size higher than 0.2 μm; 2 = fraction with size below 0.2 µm and MW higher than 8 kDa; 3 = fraction with MW between 8 and 5 kDa; 4 = fraction with MW between 5 and 1 kDa; 5 = fraction with MW lower than 1 kDa) obtained from 1 L rice protein byproduct fermentation with strains LrC1122 (A1 to A5), Lp82 (B1 to B5), and LrPRRH (C1 to C5). N.D.: not detectable. Means followed by the same letter did not differ significantly (Tukey’s test, *p* > 0.05).

The lyophilized fractions R0.2, R8, R5, R1, and P1, obtained by membrane filtration of microbial hydrolysates, were analyzed for in vitro antioxidant, antihypertensive, and anti-tyrosinase activities (Figure 2) and for cell-based anti-inflammatory, proliferation, elastin synthesis, and collagen I synthesis effects (Table 3). All samples showed antioxidant activity (expressed as mg AA eq/g of proteins), which varied with each fraction’s MW (Figure 2A). In particular, LrC1122-strain peptides with MW < 1 kDa (A5 fraction) seemed to display the greatest antioxidant activity. In fact, there was 48% more activity per weight (21.06 mg AA eq/g) in this fraction than in the total sample (14.14 mg AA eq/g). Similarly, LrPRRH-strain antioxidant peptides were mainly concentrated in the low-MW fractions, with a 102% (27.65 mg AA eq/g) and 75% (23.97 mg AA eq/g) activity increase for C4 (MW between 5 and 1 kDa) and C5 (MW < 1 kDa) fractions, respectively, compared to the total sample (13.67 mg AA eq/g). In the Lp82 strain, the three fractions with MWs below 8 kDa had similar values and demonstrated the highest activity. The average increase was about 27% with respect to the total sample (13.47, 13.42, 13.28, and 10.57 mg AA eq/g for B3, B4, and B5 samples and the total sample, respectively). Considered together, these results indicate a relationship between the antioxidant activity of rice protein hydrolyzed by the three LAB strains and the MW distribution of the hydrolysis products: the higher activity occurs in the lower MW fractions. Similar results were previously obtained after hydrolysis of the same substrate with multiple enzymes or by fermentation with *Bacillus* spp. [4,5,6]. On the other hand, another study found no relationship between antioxidant activity and the MW of peptides obtained from enzymatic hydrolysis of rice endosperm proteins [3]. This contrasting finding might be due to the different composition of the substrate and/or the differences in proteolytic specificity of different enzymes and microbial systems. Different systems could influence the length, amino acid composition, and sequence of the peptides obtained, and the resulting antioxidant (or other bioactive) properties. 

The same factors could be responsible for the significant differences in the maximum values of antioxidant activity obtained in this experiment by LAB fermentation (21.06, 13.47, and 27.65 mg AA eq/g for LrC1122, LrPRRH, and Lp82 strains, respectively) compared to the most active fractions isolated after enzymatic hydrolysis of the same substrate which were, respectively, P1 for Protamex and Alcalase (610 and 500 mg AA eq/g, respectively), and R1 for Neutrase (120 mg AA eq/g); see [6]. 

The antihypertensive activity assay showed that all the fractions, with the exception of A1 and C5, exerted some ACE-inhibitory activity (Figure 2B). The lowest IC50 values of 4.43 and 4.85 mg/L were found in fractions B5 and A5, respectively. In fact, these IC50 values were significantly lower than the other fractions. Previous studies suggested that small peptides are mainly responsible for the ACE-inhibitory activity of hydrolysates [6,31,32]. Our results confirmed this finding since fractions containing low MW peptides, such as fractions A5 and B5 (MW < 1 kDa) and fractions A4 and B4 (MW between 1 and 5 kDa), showed the highest ACE-inhibitory activity. Wang et al. [33] found that the low MW fraction (MW < 4 kDa) of a tryptic hydrolysate of rice bran proteins showed the highest ACE-inhibitory activity. Similarly, the hydrolysis (by Neutrase or Protamex) of a protein-rich byproduct of the rice-starch industry as well as the hydrolysis (by Protamex) of rice bran albumin resulted in low-MW fractions which were more active than higher MW fractions [6,34]. The present data suggest that hydrolysis of rice proteins by LAB is a suitable method for producing ACE-inhibitory peptides. In fact, some of the isolated fractions (i.e., A5 and B5) showed an inhibitory potency 30 times higher than the most active fractions isolated after enzymatic hydrolysis of the same substrate [6]. 

Samples obtained from all three in vitro treatments showed significant anti-tyrosinase activity, with the maximum in fractions with MW below 1 kDa (249.6, 227.8, and 190.6 mg of kojic acid (KA) equivalent per g of protein in samples A5, B5, and C5, respectively; Figure 2C). The differences in anti-tyrosinase activity between A5 and B5 and the other samples were statistically significant. The fractions with a particle size higher than 0.2 μm (A1, B1, and C1) and those with MW higher than 8 kDa (A2, B2, and C2) seemed to have the lowest anti-tyrosinase capacity (on average 9.0 and 4.2 mgKAeq/g, respectively; Figure 2C), while those with MW between 5 and 8 kDa (A3, B3, and C3) and those with MW between 1 and 5 kDa (A4, B4, and C4) showed increasing anti-tyrosinase activity as a function of increasing MW (on average 57.2 and 107.7 mgKAeq/g, respectively; Figure 2C). These values were similar or exceeded those obtained from a study in which a similar rice-starch byproduct was digested with commercial proteases and fractionated with the same technique [6]. The authors reported anti-tyrosinase activity between 1 and 32 mgKAeq/g prot, and observed that anti-tyrosinase capacity was generally higher in lower-MW peptide fractions obtained after Protamex, Neutrase and Alcalase treatments. Anti-inflammatory activity was assessed by a BL cell-based assay using fractions at the concentrations reported in Table 3 (columns A). Relevant bioactivity was found for A1 and C4 fractions in which cells treated with samples at dilution 1:10 (concentrations of 0.31 mg/mL and 0.02 mg/mL, respectively, Table 3) showed less reporter-gene transcription than the control, without showing severe cytotoxicity (% viability of 71.5 and 69.9, respectively). In particular, both A1 and C4 samples were able to decrease TNFα-induced inflammation by 20%. Although samples B2 and C1 decreased the inflammation level by 80% and 40%, respectively, they showed a high cytotoxicity of >75% (Table 3). The bioactivity identified in fractions A1 and C4 could be ascribed to the presence in rice glutelin storage proteins of a specific peptide sequence (arginine–glycine–aspartic acid, RGD), which has previously been reported to have anti-inflammatory and other health properties [12]. In fact, some fractions with anti-inflammatory properties were also identified in a previous work which performed enzymatic digestion of the same substrate [6]. However, to corroborate this hypothesis, further studies and a full characterization of the fractions are required. 

To measure proliferation, elastin synthesis, and collagen I synthesis effects, samples A, B, and C were diluted to a protein concentration of about 5–100 mg/L (Table 3, columns B) and tested in the Normal Human Fibroblasts (NHF) monolayer culture system. Only the A5 fraction was found to be toxic, with a final viability of 0.49%. All the other fractions displayed viabilities higher than 65%. Additionally, only the C3 fraction showed an effect on fibroblast proliferation, with a viability of 106.07% at 57.20 mg/L concentration. Samples with the highest viability for each fraction were used to estimate the expression of elastin and collagen I. Firstly, the variation of 166.58% in elastin expression for vitamin C-exposed cells (compared to untreated cells) validated the protocol and the results. In addition, the final values revealed a significant variation in elastin expression for fibroblasts exposed to the A2 fraction (+ 112.32% for 100.47 mg/L concentration) and B1 fraction (+ 145.68% for 3.14 mg/L concentration). A lesser effect was detected for most of the other samples, with variations ranging from 4.22% (C1 fraction) to 39.18% (B2 fraction), while the B3 and C3 fractions had no effect on elastin expression. The A2, A4, and C3 fractions increased collagen I expression by 67.13%, 43.95%, and 76.51%, respectively, compared to untreated cells. Finally, the B5 fraction generated the highest expression of collagen I, with an increase of 218%; all the other samples had less or no effect on collagen I synthesis. Indeed, the A2, A4, B1, and C3 samples led to a higher expression of elastin and collagen I, suggesting a possible anti-aging effect. This effect, not previously reported for rice peptides, might lead to the exploitation of this industrial byproduct in the cosmetic field that has already integrated rice hydrolysates in the formulation of many commercial health products.

## 4. Conclusions

This research identified three proteolytically active LAB strains and a process to release peptide fractions exhibiting health-related bioactivity from an industrial rice-starch byproduct. In vitro antioxidant, antihypertensive, and anti-tyrosinase activities were verified, as well as an anti-aging effect on human fibroblasts and anti-inflammatory activity on human embryonic kidney HEK293 cells. In comparison to the results of a previous study, where the same substrate underwent enzymatic digestion, some of the final fractions showed higher antihypertensive and anti-tyrosinase activities, but reduced antioxidant properties. Besides, some of the fractions increased the expression of elastin and collagen I in the NHF culture system, suggesting a potential anti-aging effect, not previously reported for rice peptides. Overall, these results indicate that microbial fermentation can be an efficient tool for the recovery of bioactive compounds from protein-containing waste or byproducts, comparable to the enzymatic proteolytic approach.

The isolated peptide fractions might be suitable for applications in the nutraceutical, functional food, and cosmetic fields. Furthermore, this biotechnological process harnessing microbial bioconversion could be a useful model for the exploitation of other protein-containing substrates currently treated as byproducts (or waste) by the food industry.

## Figures and Tables

**Table 1:** pH dynamics, number of viable Lactic Acid Bacteria (LAB) cells, protein concentration, and antioxidant activity by ABTS assay of rice protein byproduct (BR) not inoculated (control samples), and inoculated with LAB strains LrC1122, Lp82 and LrPRRH in three different experiments (50 mL scale): standard (BR); with fermentation substrate (BR with FS); with BR5X and addition of FS (BR5X with FS). Means with same letter were not significantly different (*p* > 0.05). ^1^ (Log CFU/g); ^2^ (g/L); ^3^ AA = antioxidant activity (mg ascorbic acid (AA) eq/L).

	BR				BR with FS				BR5X with FS			
	0 h	24 h	48 h	72 h	0 h	24 h	48 h	72 h	0 h	24 h	48 h	72 h
**Control**												
pH	5.65 ± 0.02 ^ij^	5.12 ± 0.03 ^l^	7.10 ± 0.05 ^f^	7.98 ± 0.03^a^	5.49 ± 0.04^a^	4.19 ± 0.02 ^c^	4.00 ± 0.01 ^d^	3.84 ± 0.03 ^ef^	5.52 ± 0.03 ^a^	4.36 ± 0.05 ^c^	4.18 ± 0.02 ^d^	4.03 ± 0.02 ^e^
LAB cells **^1^**	7.29 ± 0.10 ^h^	7.82 ± 0.05 ^g^	8.18 ± 0.08 ^def^	8.53 ± 0.09 ^b^	6.89 ± 0.07 ^h^	8.47 ± 0.05 ^d^	8.87 ± 0.08 ^c^	7.69 ± 0.09 ^e^	8.21 ± 0.09 ^h^	8.85 ± 0.07 ^fg^	8.97 ± 0.08 ^f^	9.12 ± 0.05 ^ef^
Proteins **^2^**	0.47 ± 0.01 ^l^	0.56 ± 0.01^i^	0.65 ± 0.00 ^h^	1.16 ± 0.00 ^b^	1.27 ± 0.01 ^h^	3.33 ± 0.03 ^c^	4.05 ± 0.01 ^a^	4.15 ± 0.06 ^a^	1.13 ± 0.02 ^j^	2.32 ± 0.01 ^g^	3.76 ± 0.08 ^d^	5.02 ± 0.04 ^b^
AA ^3^	12.25 ± 0.69 ^h^	22.42 ± 0.68^d^	15.64 ± 0.00 ^fgh^	92.01 ± 2.23 ^b^	16.54 ± 0.49 ^h^	39.85 ± 0.97 ^e^	41.46 ± 0.64 ^de^	47.80 ± 1.30 ^c^	12.51 ± 0.51 ^g^	30.10 ± 0.69 ^f^	54.99 ± 1.64 ^c^	56.54 ± 0.88 ^bc^
**LrC1122**												
pH	5.59 ± 0.01 ^j^	5.82 ± 0.02 ^h^	7.75 ± 0.05 ^c^	7.95 ± 0.02 ^ab^	5.34 ± 0.05 ^b^	3.78 ± 0.01 ^fg^	3.69 ± 0.03 ^h^	3.60 ± 0.02 ^i^	5.44 ± 0.01 ^b^	3.66 ± 0.02 ^j^	3.77 ± 0.03 ^hi^	3.83 ± 0.05 ^gh^
LAB cells **^1^**	9.38 ± 0.09 ^a^	8.28 ± 0.07 ^bcde^	8.35 ± 0.08 ^bcd^	8.52 ± 0.10 ^bc^	9.11 ± 0.10 ^bc^	9.39 ± 0.08 ^ab^	8.95 ± 0.11 ^c^	7.39 ± 0.10 ^f^	9.45 ± 0.09 ^cd^	9.81 ± 0.10 ^b^	9.59 ± 0.07 ^bcd^	8.21 ± 0.08 ^h^
Proteins **^2^**	0.54 ± 0.02 ^ij^	0.90 ± 0.01^e^	0.80 ± 0.00 ^g^	1.04 ± 0.01^c^	1.04 ± 0.03 ^i^	2.89 ± 0.05 ^f^	3.02 ± 0.02 ^de^	3.39 ± 0.04 ^c^	1.34 ± 0.05 ^i^	3.76 ± 0.02 ^d^	4.61 ± 0.05 ^c^	5.54 ± 0.03 ^a^
AA ^3^	13.01 ± 0.93 ^gh^	16.30 ± 0.87 ^fgh^	22.10 ± 0.38 ^de^	104.38 ± 3.15^a^	13.64 ± 0.49 ^i^	34.91 ± 1.04 ^f^	34.37 ± 0.85 ^f^	66.49 ± 0.93 ^b^	15.22 ± 0.70 ^g^	44.76 ± 0.70 ^e^	53.00 ± 1.17 ^cd^	60.19 ± 1.15 ^a^
**Lp82**												
pH	5.68 ± 0.02 ^i^	6.42 ± 0.03 ^g^	7.59 ± 0.01 ^d^	7.97 ± 0.02 ^a^	5.37 ± 0.01 ^b^	3.86 ± 0.02 ^e^	3.80 ± 0.02^efg^	3.68 ± 0.01 ^hi^	5.45 ± 0.01 ^ab^	3.73 ± 0.03 ^ij^	3.81 ± 0.02 ^hi^	3.91 ± 0.03 ^f^
LAB cells **^1^**	9.39 ± 0.07 ^a^	8.36 ± 0.08 ^bcd^	8.35 ± 0.05 ^bcd^	8.23 ± 0.08 ^cde^	9.14 ± 0.08 ^bc^	9.53 ± 0.10 ^a^	8.22 ± 0.07 ^d^	7.07 ± 0.09 ^gh^	9.76 ± 0.10 ^b^	9.65 ± 0.09 ^bc^	9.55 ± 0.07 ^bcd^	8.59 ± 0.08 ^g^
Protein **^2^**	0.53 ± 0.01^jk^	0.86 ± 0.01 ^f^	1.04 ± 0.01^c^	1.15 ± 0.01^b^	1.09 ± 0.02 ^i^	2.43 ± 0.03 ^g^	2.91 ± 0.03 ^f^	3.53 ± 0.02 ^b^	0.98 ± 0.04 ^k^	2.22 ± 0.04 ^g^	3.27 ± 0.07 ^e^	4.58 ± 0.03 ^c^
AA ^3^	12.19 ± 0.70 ^h^	17.17 ± 0.6 ^efgh^	52.95 ± 1.65 ^c^	103.61 ± 4.34 ^a^	17.94 ± 0.56 ^h^	34.05 ± 1.12 ^f^	35.98 ± 0.32 ^f^	42.75 ± 0.32 ^d^	13.51 ± 0.70 ^g^	44.81 ± 1.53 ^e^	51.01 ± 0.84 ^d^	59.30 ± 0.51 ^ab^
**LrPRRH**												
pH	5.59 ± 0.05 ^j^	5.23 ± 0.03 ^k^	7.37 ± 0.02 ^e^	7.88 ± 0.02 ^b^	5.33 ± 0.02 ^b^	3.96 ± 0.05 ^d^	3.87 ± 0.03 ^e^	3.73 ± 0.01 ^gh^	5.43 ± 0.03 ^b^	3.82 ± 0.02 ^h^	3.85 ± 0.01 ^fgh^	3.90 ± 0.02 ^fg^
LAB cells **^1^**	9.53 ± 0.10 ^a^	8.02 ± 0.05 ^efg^	7.86 ± 0.10 ^g^	7.90 ± 0.07 ^fg^	9.04 ± 0.09 ^c^	8.95 ± 0.07 ^c^	8.52 ± 0.05 ^d^	7.28 ± 0.08 ^fg^	9.40 ± 0.08 ^cde^	9.34 ± 0.10 ^de^	9.41 ± 0.07 ^cde^	10.76 ± 0.05 ^a^
Proteins **^2^**	0.50 ± 0.01 ^k^	0.98 ± 0.01^d^	1.17 ± 0.01 ^b^	1.82 ± 0.02 ^a^	1.07 ± 0.03 ^i^	2.92 ± 0.02 ^ef^	3.10 ± 0.02 ^d^	3.32 ± 0.04 ^c^	0.93 ± 0.03 ^k^	1.91 ± 0.02 ^h^	3.10 ± 0.04 ^f^	4.50 ± 0.05 ^c^
AA ^3^	12.80 ± 0.38 ^gh^	20.35 ± 0.76 ^def^	17.50 ± 0.8 ^defg^	91.80 ± 0.99 ^b^	18.48 ± 0.19 ^h^	30.08 ± 0.19 ^g^	35.12 ± 0.37 ^f^	79.80 ± 0.56 ^a^	15.50 ± 1.34 ^g^	50.56 ± 1.85 ^d^	56.32 ± 1.57 ^bc^	59.30 ± 1.38 ^ab^

**Table 2:** Code, filtration loss (%), concentration factor (mL/mL), and dry matter (g/L) of fractions R0.2 (particle size > 0.2 µm), R8 (size < 0.2 µm and MW > 8 kDa), R5 (MW = 8–5 kDa), R1 (MW = 1–5 kDa), and P1 (MW < 1 kDa) obtained from 1 L fermentation samples of rice protein byproduct, with lactic acid bacteria strains LrC1122, Lp82, and LrPRRH.

Sample	Fraction	Code	Filtration Loss (%)	Concentration Factor (mL/mL)	Dry Matter (g/L)
**LrC1122**	R0.2	A1	0.41	47.86	46.48
	R8	A2	0.14	16.25	65.00
	R5	A3	0.97	6.22	42.29
	R1	A4	1.01	8.10	37.26
	P1	A5	/	/	27.98
**Lp82**	R0.2	B1	0.32	14.14	19.13
	R8	B2	0.43	33.04	79.22
	R5	B3	0.35	9.02	46.34
	R1	B4	5.49	8.13	41.33
	P1	B5	/	/	28.25
**LrPRRH**	R0.2	C1	0.68	42.89	41.94
	R8	C2	0.23	8.88	56.11
	R5	C3	2.80	5.83	33.33
	R1	C4	1.54	8.38	32.75
	P1	C5	/	/	26.80

**Table 3:** Cytotoxicity effects and anti-inflammatory activity (A), and proliferation, elastin synthesis and collagen I synthesis effects (B), of fractions 1 to 5 (1 = fraction with particle size higher than 0.2 μm; 2 = fraction with size below 0.2 µm and MW higher than 8 kDa; 3 = fraction with MW between 8 and 5 kDa; 4 = fraction with MW between 5 and 1 kDa; 5 = fraction with MW lower than 1 kDa), obtained from 1 L rice protein byproduct fermentation with strains LrC1122 (samples A1 to A5), Lp82 (samples B1 to B5) and LrPRRH (samples C1 to C5). (1:10) indicates a ten times dilution of the sample; N.C. indicates negative control; N.D. indicates not detectable. ** conc = concentration; * i nh = inhibition; ^†^ Evol = evolution; ^1^ %Cytotoxicity (% viability); ^††^ anti-infl = anti-inflammatory.

	A			B					
Fraction	Conc ** (mg/mL)	Cytotox ^1^	Anti-Infl Activity ^††^	Conc ** (mg/L)	Viability (%)	Elastin Conc ** (pg/mL)	Evol^†^ (%)	Collagen I Conc ** (ng/mL)	Evol ^†^ (%)
**N.C.**		100	1.00 ± 0.05		100.00	21.14	/	2.03	/
**A1**	0.31	71.5	0.8 ± 0.01 (20% inh *)	15.73	93.48	20.81	+26.72	34.38	+8.87
**A2**	2.16	3.4	N.D.	100.47	88.98	34.86	+112.32	52.78	+67.13
**A3**	0.20	0.8	N.D.	59.69	96.45	20.15	+22.70	38.55	+22.08
**A4**	0.03	81.8	0.89 ± 0.08	49.20	96.81	19.75	+20.29	45.46	+43.95
**A5**	0.06	0.3	0.52 ± 0.45	32.72	0.49	/	/	/	/
**B1**	0.44	8.4	N.D.	3.14	98.88	40.34	+145.68	33.36	+5.65
**B2**	2.16	9.9	0.20 ± 0.01	3.52	94.65	22.85	+39.18	35.78	+13.31
**B3**	0.20	8.2	N.D.	30.75	88.26	16.19	−1.41	25.31	−19.86
**B4**	0.09	3.5	N.D.	46.30	94.29	17.90	+9.04	24.64	−21.97
**B5**	0.07	0.2	1.2 ± 0.3	54.13	85.66	13.63	−35.51%	6.47	+217.89%
**C1**	0.70	24.7	0.60 ± 0.1	3.63	93.75	17.11	+4.22	34.99	+10.79
**C2**	2.08	0.9	N.D.	6.80	93.75	22.13	+34.76	38.39	+21.57
**C3**	0.15	0.6	N.D.	57.20	106.07	18.83	+14.67	55.74	+76.51
**C3 (1:10)**	/	/	/	5.72	102.74	11.24	−31.55	27.32	−13.51
**C4**	0.02	69.9	0.8 ± 0.1 (20% inh *)	31.56	100.13	20.15	+22.70	38.84	+22.98
**C4 (1:10)**	/	/	/	3.16	101.93	19.22	+17.08	27.44	−13.10
**C5**	0.11	0.2	1.0 ± 0.5	44.53	65.44	4.50	−78.73%	0.00	/

## Supplementary Materials

Supplementary materials can be found at https://www.mdpi.com/2076-2607/8/7/986/s1.

## Abbreviations

AA	ascorbic acid
AAeq	ascorbic acid equivalents
ABTS	2,2,-azino-bis(3-ethylbenz-thiazoline-6-sulfonic) acid
BR	rice protein byproduct
BR5X	rice protein byproduct containing the solid fraction concentrated five times
BSA	bovine serum albumin
DPPH	1,1-DiPhenyl-2-PicrylHydrazyl
FS	fermentation substrate
KA	kojic acid
LAB	lactic acid bacteria
LbPRBU	Lactobacillus bulgaricus PRBU
Lclbcd	Lactobacillus casei lbcd
LfMR13	Lactobacillus fermentum MR13
LfPRFE	Lactobacillus fermentum PRFE
Lp325	Lactobacillus plantarum 325
Lp6BHI	Lactobacillus plantarum 6BHI
Lp82	Lactobacillus plantarum 82
LpPRPL	Lactobacillus plantarum PRPL
LrC1122	Lactobacillus rhamnosus C1122
LrC249	Lactobacillus rhamnosus C249
LrPRRH	Lactobacillus rhamnosus PRRH
MW	molecular weight
PAGE	polyacrylamide gel electrophoresis
SDS	sodium dodecyl sulfate.
