# Supplementary material for: Microbial Fermentation of Industrial Rice-Starch Byproduct as Valuable Source of Peptide Fractions with Health-Related Activity
Source: Microorganisms. 2020 Jun 30;8(7):986. doi: 10.3390/microorganisms8070986 (PMC7409224; doi:10.3390/microorganisms8070986)
Supplement: Supplementary file 1 [file microorganisms-08-00986-s001.pdf]

Figure S1. SDS-PAGE 14% of the initial rice-starch byproduct (BR): 1) total sample; 2) solid fraction; 3) liquid phase (in a 25:1 ratio with respect to total and pellet samples). Mk: MW marker.

Figure S2. Protein concentration (g/L, A) and antioxidant activity measured by ABTS (mg AA eq/L, B) and DPPH (mg AA eq/L, C) assays, of: rice protein byproduct (BR) control at time 0 (diagonal lines); BR and BR inoculated with 11 different lactic acid bacterial strains (LfMR13, Lp6BHI, LrC1122, Lp325, Lp82, Lclbcd, LrC249, LfPRFE, LpPRPL, LrPRRH, LbPRBU), at 72 h incubation time (gray). Means followed by the same letter did not differ significantly (Tukey test, $p>0.05$).

Figure S3. Tricine SDS-PAGE of BR at time 0 (T0) and after 72 h incubation with LAB strains LrC1112 (A), Lp82 (B) and LrPRRH (C), in 1 L large-scale experiments. Mk: MW marker.

Figure S4. PCR agarose gel of LAB after a 72 h fermentation in BR+FS substrate
X taq = Not inoculated + Taq polymerase; X hifi = Not inoculated + Platinum HI-FI polymerase; LCtaq = LrC1122 + Taq polymerase; LChifi = LrC1122 + Platinum HI-FI polymerase; L8taq = Lp82 + Taq polymerase; L8hifi = Lp82 + Platinum HI-FI polymerase; LPtaq = LrPRRH + Taq polymerase; LPhifi = LrPRRH + Platinum HI-FI polymerase.

Figure S1

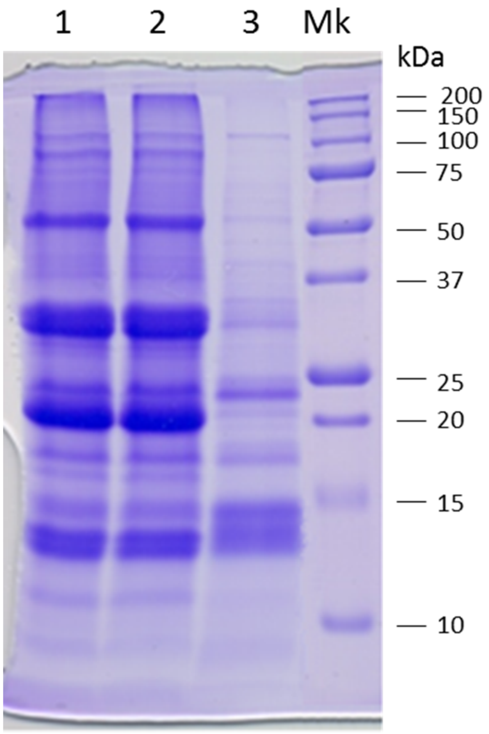

Figure S2

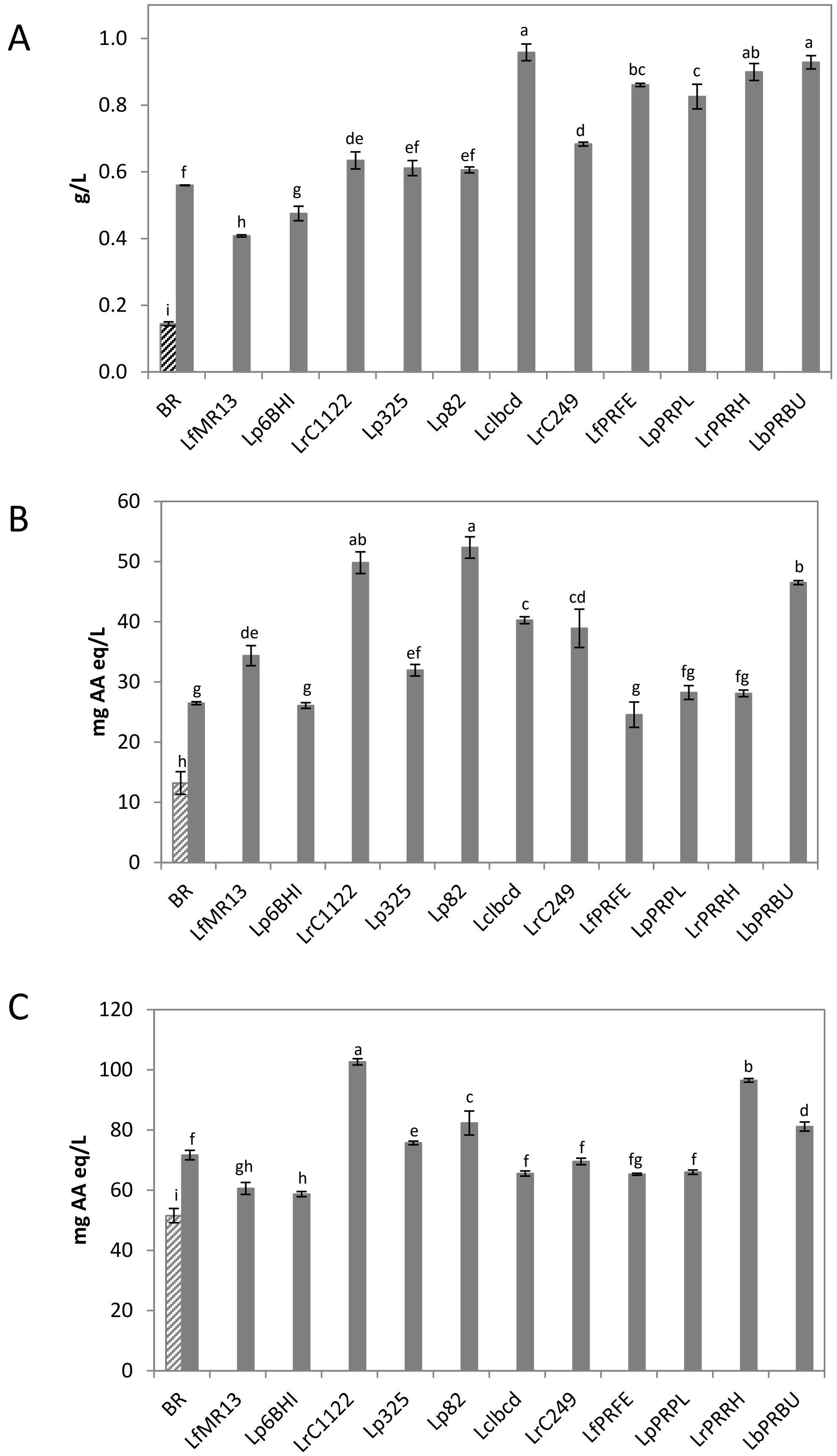
A
g/L
0.0
0.2
0.4
0.6
0.8
1.0
i
f
h
g
de
ef
ef
a
d
bc
c
ab
a
BR
LfMR13
Lp6BHI
LrC1122
Lp325
Lp82
Lclbcd
LrC249
LfPRFE
LpPRPL
LrPRRH
LbPRBU
B
mg AA eq/L
0
10
20
30
40
50
60
h
g
de
g
ab
ef
a
c
cd
g
fg
fg
b
BR
LfMR13
Lp6BHI
LrC1122
Lp325
Lp82
Lclbcd
LrC249
LfPRFE
LpPRPL
LrPRRH
LbPRBU
C
mg AA eq/L
0
20
40
60
80
100
120
i
f
gh
h
a
e
c
f
f
fg
f
b
d
BR
LfMR13
Lp6BHI
LrC1122
Lp325
Lp82
Lclbcd
LrC249
LfPRFE
LpPRPL
LrPRRH
LbPRBU

Figure S3.

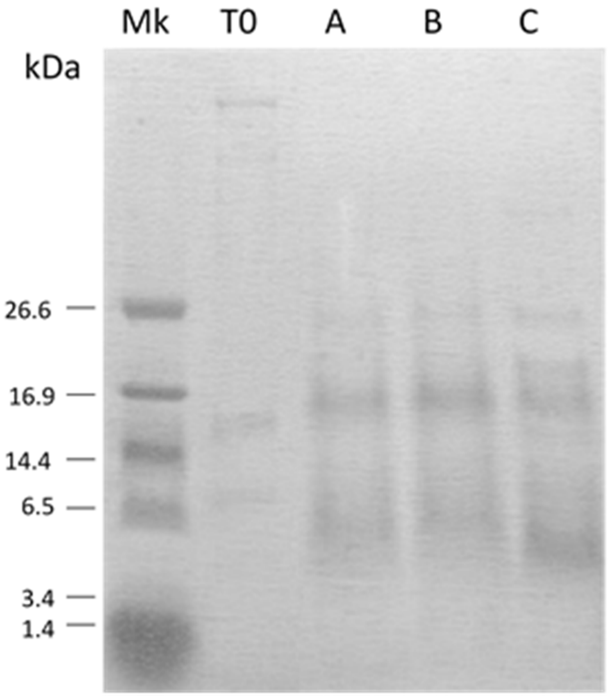

Figure S4

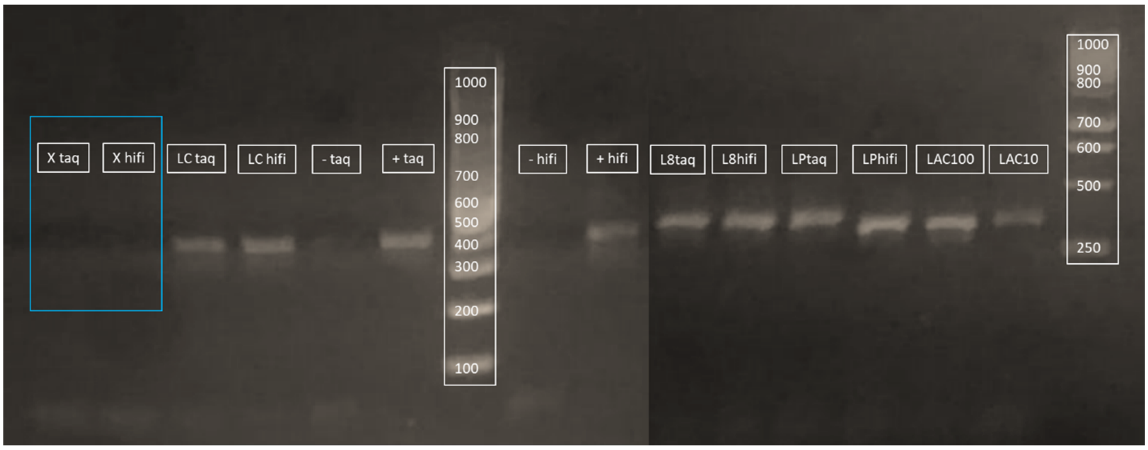

Table S1. pH dynamics, number of viable LAB cells (Log CFU/g), protein concentration (g/L), and antioxidant activity (mg AA eq/L) measured by ABTS assay, at incubation times 0, 24, 48 and 72 h, of BR incubated with LAB strains LrC11122, Lp82 and LrPRRH in 1L large-scale experiments.

	0h	24h	48h	72h
**LrC11122**				
pH	5.10 ± 0.01	3.72 ± 0.04	3.62 ± 0.03	3.63 ± 0.02
LAB cells [1]	9.38 ± 0.09	8.28 ± 0.07	8.35 ± 0.08	8.52 ± 0.10
Proteins [2]	0.88 ± 0.04	2.51 ± 0.02	3.32 ± 0.04	3.58 ± 0.01
AA [3]	22.19 ± 0.68	41.67± 0.57	49.61 ± 0.68	50.59 ± 0.50
**Lp82**				
pH	5.22 ± 0.01	3.91 ± 0.03	3.67 ± 0.02	3.65 ± 0.01
LAB cells [1]	9.39 ± 0.07	8.36 ± 0.10	8.35 ± 0.08	8.23 ± 0.07
Proteins [2]	0.73 ± 0.01	2.41 ± 0.03	3.40 ± 0.03	4.25 ± 0.01
AA [3]	20.45 ± 0.57	42.10 ± 0.82	42.65 ± 0.86	44.93 ± 0.33
**LrPRRH**				
pH	5.20 ± 0.02	3.89 ± 0.01	3.81 ± 0.04	3.87 ± 0.03
LAB cells [1]	9.53 ± 0.10	8.02 ± 0.07	7.86 ± 0.08	7.90 ± 0.07
Proteins [2]	0.79 ± 0.03	2.47 ± 0.02	3.07 ± 0.03	3.93 ± 0.01
AA [3]	20.67 ± 0.75	41.45 ± 0.50	51.24 ± 0.50	53.75 ± 0.98

[1] Viable LAB cells (Log CFU/g)

[2] Protein concentration (g/L)

[3] Antioxidant activity (mg AA eq/L)
